# Unique C2V3 Sequence in HIV-1 Envelope Obtained from Broadly Neutralizing Plasma of a Slow Progressing Patient Conferred Enhanced Virus Neutralization

**DOI:** 10.1371/journal.pone.0046713

**Published:** 2012-10-03

**Authors:** Rajesh Ringe, Lipsa Das, Ipsita Choudhary, Deepak Sharma, Ramesh Paranjape, Virander Singh Chauhan, Jayanta Bhattacharya

**Affiliations:** 1 Department of Molecular Virology, National AIDS Research Institute, Pune, India; 2 International Center for Genetic Engineering and Biotechnology, New Delhi, India; Harvard Medical School, United States of America

## Abstract

Broadly neutralizing antibodies to HIV-1 usually develops in chronic infections. Here, we examined the basis of enhanced sensitivity of an *env* clone amplified from cross neutralizing plasma of an antiretroviral naïve chronically infected Indian patient (ID50 >600-fold higher compared to other autologous *env* clones). The enhanced autologous neutralization of pseudotyped viruses expressing the sensitive envelope (Env) was associated with increased sensitivity to reagents and monoclonal antibodies targeting distinct sites in Env. Chimeric viruses constructed by swapping fragments of sensitive Env into resistant Env backbone revealed that the presence of unique residues within C2V3 region of gp120 governed increased neutralization. The enhanced virus neutralization was also associated with low CD4 dependence as well as increased binding of Env trimers to IgG1b12 and CD4-IgG2 and was independent of gp120 shedding. Our data highlighted vulnerabilities in the Env obtained from cross neutralizing plasma associated with the exposure of discontinuous neutralizing epitopes and enhanced autologous neutralization. Such information may aid in Env-based vaccine immunogen design.

## Introduction

The remarkable diversity and compactness of heavily glycosylated Human Immunodeficiency virus type 1 (HIV-1) envelope (Env) glycoprotein restricts immune evasion by neutralizing antibodies, thereby posing impediment in generating a strong and sustained neutralizing antibody response. Individuals with chronic HIV-1 infection in absence of antiretroviral therapy (ART) tend to develop cross-neutralizing antibodies over a period of years [1], [2], [3], [4], [5], [6], [7]. More recently, Lynch *et al* [8] by examining different cohorts demonstrated that the breadth and potency of CD4 binding site antibodies (CD4bs) such as VRC01 like antibodies often takes years to develop. It is widely assumed that a vaccine to HIV-1 ideally should elicit neutralizing antibodies (NAb) in addition to other immune responses to successfully prevent infection. Immunogens tested towards eliciting broad neutralizing antibodies (bNAbs) responses however yielded only little success [9], [10], [11]. Because of the complexity and antigenic diversity of epitopes on the primary viruses the NAbs produced during infection cycle are not able to cover the vast spectrum of neutralizing epitopes at the population level. Secondly, HIV-1 is known to adopt multiple mechanisms to escape from the immune response through faster evolution of highly variable envelope protein [12], [13]. These challenges have hindered the design of appropriate immunogens that would elicit broad NAbs. Recent discoveries of a number of broad and potent human neutralizing monoclonal antibodies to HIV-1 [14], [15], [16] from rare donors have highlighted directions in designing immunogens that would elicit such antibodies. Though majority of the individuals elicit type specific response mostly directed to variable domains [17], [18], [19], [20] around 20–30% infected people mount potent and cross neutralizing response capable of inhibiting diverse strains of HIV-1 [4], [5], [21], [22]. Identification of the targets of such broadly cross neutralizing (BCN) antibodies is crucial; as these epitopes likely represent novel targets for rationale Env based vaccine design. Thus, an immunogen that can elicit the antibodies of both these specificities can mount severe pressure on the virus and help in widening the breadth of the response.

In the present study, we examined the relationship between genetic and neutralization properties of autologous HIV-1 Envs that modulated neutralization properties of circulating envelopes obtained from a chronically infected Indian patient whose plasma showed BCN response [23]. By constructing the chimeric Envs between sensitive and resistant *env* clones we mapped determinants in the C2V3 region which were found to modulate neutralization properties of autologous Envs amplified from this patient.

## Results

### Neutralization Sensitivity of Contemporaneous Envs to Autologous and Heterologous Plasma Antibodies

**Figure 1:**
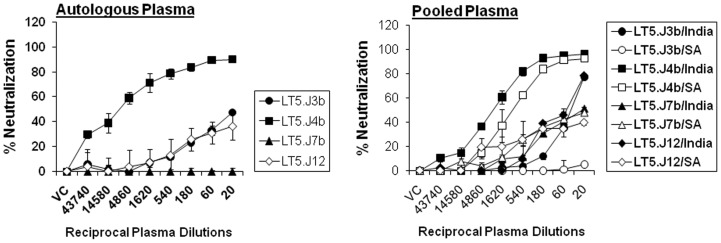
Neutralization sensitivity of pseudotyped viruses expressing NARI-LT5 envelopes to autologous and heterologous plasma antibodies. Heterologous pooled HIV-1 positive plasma samples were prepared from ten Indian and ten South African (SA) donors.

We previously reported [23] that the plasma of an ART naïve Indian patient NARI-LT5 (patient ID 991566; referred to as LT5 hereafter) who was chronically infected with HIV-1 for over eight years showed cross neutralization of several heterologous clade C and non-clade C including tier 2 and 3 Envs suggesting presence of broadly cross neutralizing antibodies (BCN). We obtained several *env* clones from plasma of this patient that showed presence of clade C and non-clade C mosaics; however only four functional Envs were obtained that gave detectable infectious titers as Env-pseudotyped viruses. The limited number of *env* clones obtained from the LT5 plasma showed genetic heterogeneity [24] and only 4/14 *env* clones were found to be functional and gave reasonable infectivity titers. Sequence analysis indicated that out of these four *env* clones, two were of pure clade C (LT5.J3b and LT5.J7b) and two were B/C recombinants (LT5.J4b and LT5.J12). First we examined the neutralization sensitivity of pseudotyped viruses expressing these four Envs to contemporaneous autologous plasma. As shown in Figure 1, except one Env, LT5.J4b (ID50 1: 12010), all the three Envs: LT5.J3b, LT5.J7b and LT5.J12 were resistant (ID50<1∶20) to the autologous plasma antibodies. Compared to other three Envs, the LT5.J4b Env showed enhanced sensitivity to autologous plasma (ID50 of 1∶12010). To further examine the susceptibility of LT5.J4b Env against heterologous HIV-1+ plasma antibodies, Env-pseudotyped virus expressing LT5.J4b Env was tested against clade C pooled plasma obtained from Indian and South African donors. As expected, LT5.J4b Env showed enhanced and comparable sensitivity to heterologous plasma pools (ID50 of 1∶2668 and 1∶930 to India and South Africa clade C pools respectively). Our data indicated that the LT5.J4b Env possessed the neutralizing epitopes that were absent on the other contemporaneous Envs and which possibly conferred neutralization escape to autologous antibodies.

### Exposure of Neutralizing Epitopes on Multiple Sites in LT5.J4b Env

**Figure 2:**
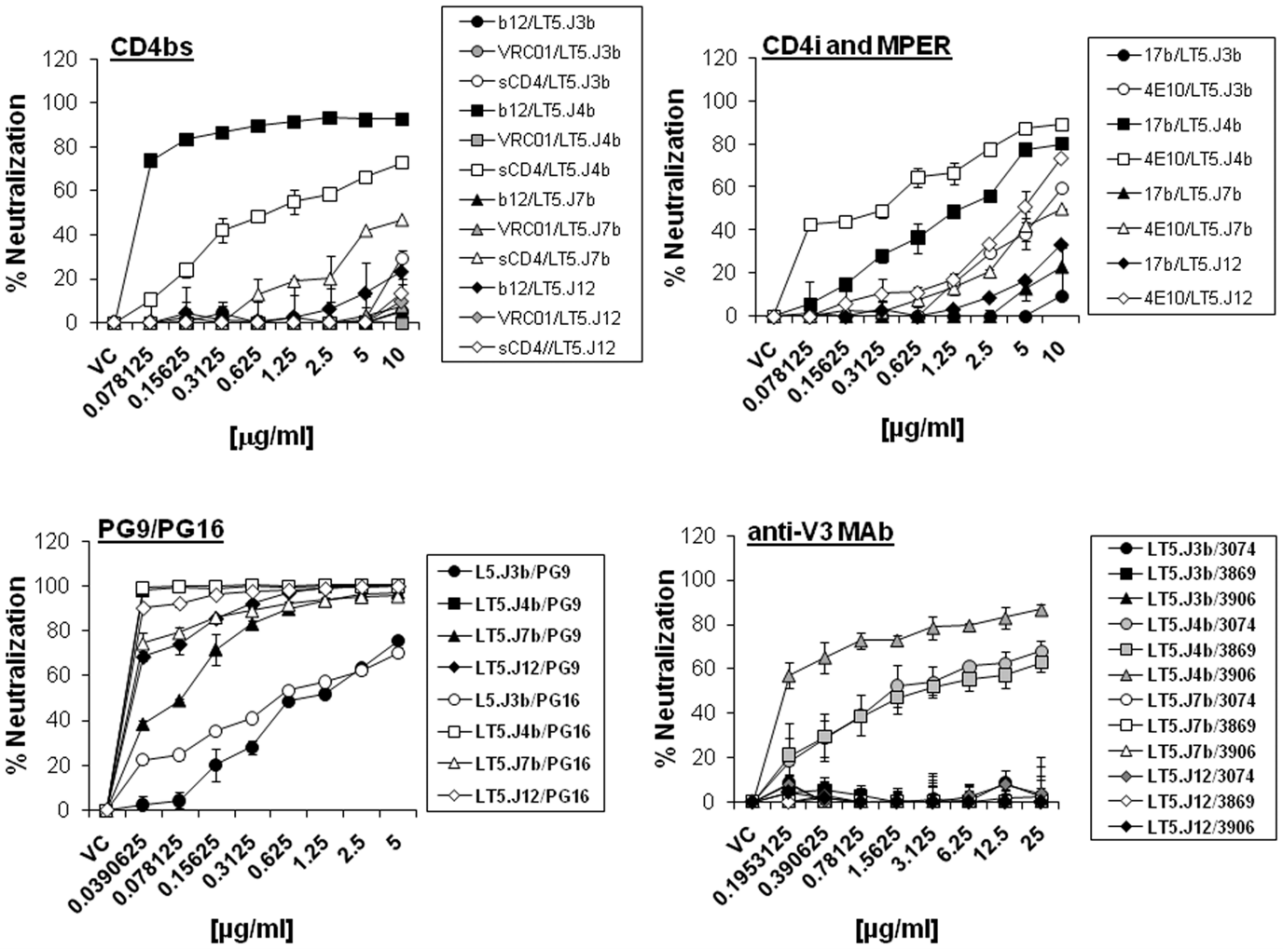
Neutralization sensitivity of pseudotyped viruses expressing four different envelopes amplified from NARI-LT5 plasma to monoclonal antibodies targeting different sites in gp120 and gp41.

Next, we examined the degree of sensitivity of these four LT5 Envs to reagents that target distinct sites in Envs, such as CD4bs (MAbs IgG1b12, VRC01 and sCD4), V3 loop (MAbs 3074 and 3869), CD4-induced epitopes/coreceptor binding site (17b MAb) and MPER (MAbs 4E10 and 2F5) [25], [26]. As shown in Figure 2, only the LT5.J4b Env showed enhanced sensitivity to IgG1b12, sCD4, anti-V3 MAbs and modestly to 17b, 4E10 and 2F5 (Figure S1). Interestingly, all the LT5 Envs were found to be resistant to VRC01 (Figure S1) suggesting lack of optimal exposure of epitopes required for VRC01 MAb susceptibility in all these Envs. Only LT5.J4b and LT5.J12 were found to be sensitive to 2F5 as opposed to LT5.J3b and LT5.J7b owing to absence of the minimal DKW motif in membrane proximal region (MPER) of the later two Envs. We have recently shown [27] that all the four LT5 Envs except LT5.J3b showed enhanced susceptibility to PG9 and PG16 MAbs that targets quaternary (QNE) epitopes made by V1V2 and V3 loops. While the relative resistance of the LT5.J3b Env was found to be due to alterations in glycan positioning and V2 loop characteristics, the LT5.J4b Env was found to be most potently neutralized by PG9 and PG16 MAbs compared to other three LT5 Envs [27]. Overall, our data suggested the following: (1) LT5.J4b Env possesses unique sequence among LT5 Envs that was responsible for the enhanced susceptibility to the neutralizing antibodies and (2) LT5.J4b has multiple neutralizing epitopes exposed which are apparently concealed in rest of the LT5 Envs.

### Unique C2V3 Sequence Conferred Enhanced Neutralization Sensitivity of LT5.J4b Env

**Figure 3:**
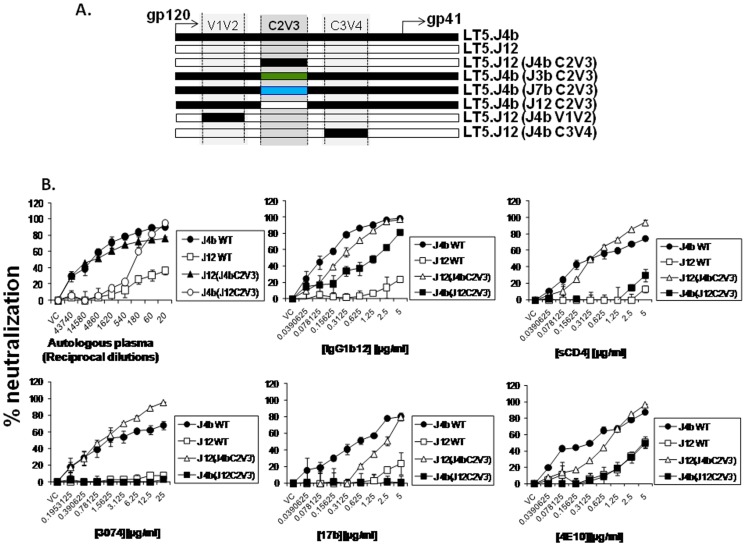
Neutralization properties of chimeric LT5 Envs. A. Construction of chimeric LT5 envelopes. B. Neutralization sensitivity of pseudotyped viruses expressing wild type and chimeric LT5 envelopes to autologous plasma and human monoclonal antibodies targeting different sites in gp120 and gp41.

**Figure 4:**
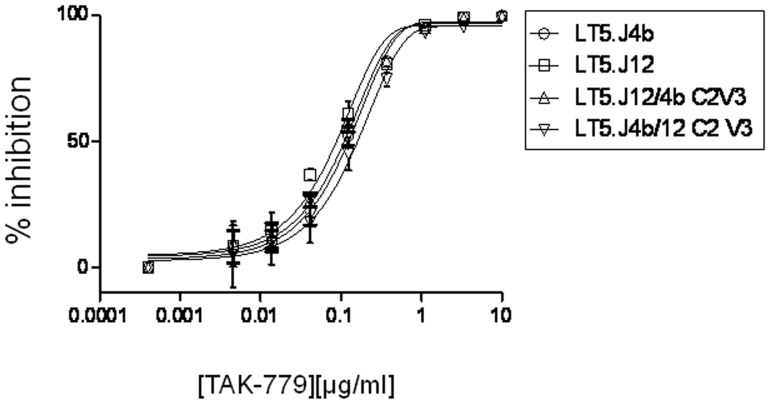
Effect of CCR5 antagonist, TAK-779 on entry inhibition of chimeric LT5 envelope in TZM-bl cells. Pseudotyped viruses expressing different chimeric LT5 envelopes were titrated against different concentrations of TAK-779. The reduction in infection with the serially diluted TAK779 was assessed by measuring RLU and indicated as % neutralization on Y-axis. Note that comparable inhibition of CCR5 mediated entry of LT5.J4b and J12 were found despite differences in their sensitivity to 17b MAb.

To dissect the sequence of the LT5.J4b Env conferring enhanced neutralization, first we swapped different segments between the LT5.J4b (sensitive) and LT5.J12 (resistant) Envs to construct chimeric Envs (Figure 3A). Pseudotyped viruses expressing the wild type and the chimeric Envs were subsequently tested for their neutralization sensitivity against autologous plasma and different MAbs. As shown in Figure 3B, pseudoviruses containing the LT5.J12 Env expressing C2V3 of the LT5.J4b Env, referred to as J12 (J4bC2V3) became comparably sensitive to autologous plasma as well as to IgG1b12, sCD4 and anti-V3 MAb (3074). Modest increase in neutralization sensitivity of this chimeric Env to 17b, 4E10 and 2F5 MAbs was also noticed. To confirm the effect of the C2V3 sequence, the C2V3 region of LT5.J4b Env was replaced with that of rest three LT5 Envs (J3b, J7b and J12) producing the reverse chimera. It was found that the pseudoviruses expressing chimeric LT5.J4b Env became resistant to autologous plasma antibodies as well as to the MAbs tested; suggesting clearly that LT5.J4b Env contained unique C2V3 sequence that primarily conferred enhanced neutralization sensitivity. Modest sensitivity of Envs containing C2V3 sequence of LT5.J4b to 17b MAb suggested relatively increased exposure of coreceptor binding sites; however no difference in their sensitivity to CCR5 antagonist, TAK-779 [28] was found (Figure 4). Our data indicated that presence of unique C2V3 sequence in LT5.J4b Env likely altered Env conformation towards greater exposure of discontinuous neutralization epitopes which allowed optimum access or binding to the neutralizing antibodies towards comprehensive interference of virus entry.

**Figure 5:**
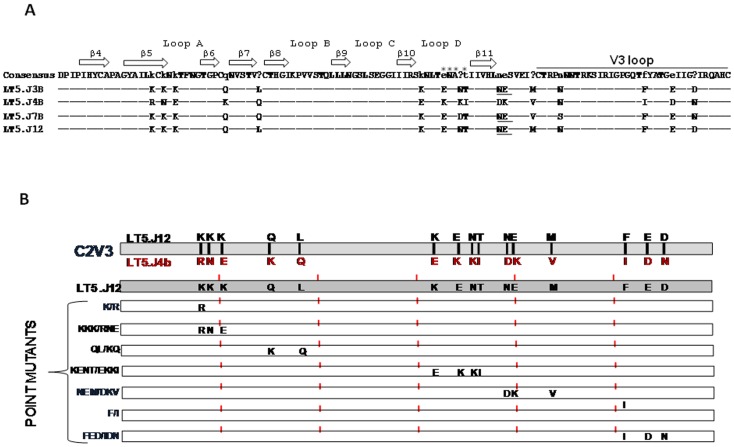
Fine mapping of C2V3 region of LT5.J4b Env. A. The alignment of C2V3 sequences of four LT5 Envs is shown. The secondary structure assignments are shown as arrows. Potential N-linked glycosylation sites are underlined. Residues of gp120 in direct contacts with CD4 are indicated by an asterisk. B. The panels of C2V3 mutant clones made to fine map the determinants in this region for neutralization sensitivity. All mutations were made in LT5.J12 Env backbone which acted as recipient Env to acquire corresponding amino acids present in LT5.J4b Envs. The mutant Envs were constructed by substitution of single amino acid or patches (combining more than one amino acid substitutions) from the sensitive LT5.J4b to LT5.J12 Env by blunt end ligation.

At this juncture, we wanted to fine map residue/s within C2V3 sequence in LT5.J4b Env to examine if there is/are any specific residue/s that possibly contributed to enhanced virus neutralization. When compared between LT5.J4b and LT5.JJ12 Envs, we found fifteen differences in amino acid residues in C2V3 region (twelve in the C2 and three in the V3 loop). Specific point substitutions were made in the LT5.J12 Env as described in Methods and pseudoviruses expressing mutant Envs were prepared. Interestingly, we did not find any specific substitution conferring sensitivity to LT5.J12 Env (Table 1). The Q240K and L246Q substitution in C2 region showed marginal but not significant increase in the sensitivity of LT5.J12 to IgG1b12 and sCD4. Our data indicated that presence of unique C2V3 sequence in the LT5.J4b Env modulated the neutralization competent structures at CD4bs and V3 loop towards enhanced neutralization susceptibility of the LT5.J4b Env. The total number of N-linked glycosylation sites in LT5.J12 Env was found to be seven compared to six in LT5.J4b Env. Removal of extra glycosylation site from LT5.J12 Env by substitution of N289D, E290K and M295V (Figure 5 and Table 1) did not alter the sensitivity of the LT5.J12 Env, thus negating any role of glycan in masking the neutralization epitopes. Overall, scanning of the entire C2V3 region by making substitutions revealed that the individual amino acid residue or a particular combination of residues was insufficient to modulate neutralization sensitivity of LT5 Env; rather it was unique sequence pattern in the C2V3 region that cooperatively modulated the sensitivity of the LT5 Envs.

**Table 1:** Neutralization sensitivity of chimeric Envs.

	µg/ml				(ReciprocalDilutions)
**LT5.J4b**	0.05	1.3	0.2	0.62	12010
**LT5.J12**	>10	>10	>10	>10	<50
**J12(J4b C2V3)**	0.15	2.2	0.2	0.5	9720
**K227R**	>10	>10	>10	>10	<50
**K227R/K229N/K231E**	>10	>10	>10	>10	<50
**Q240K/L246Q**	9.5	>10	>10	6.8	330
**K275E**	>10	>10	>10	>10	<50
**E279K**	>10	>10	>10	>10	<50
**N282K**	>10	>10	>10	>10	<50
**T283I**	>10	>10	>10	>10	<50
**K275E/ E279K/ N282K/T283I**	>10	>10	>10	>10	<50
**N289D/E290K/M295V**	>10	>10	>10	>10	<50
**F317I**	>10	>10	>10	>10	<50
**F317I/E321D/D325N**	>10	>10	>10	>10	<50

* Values indicate reciprocal dilution for LT5 plasma. Plasma dilutions less than 1∶50 required to provide 50% neutralization in TZM-bl cells are referred to as <50. The monoclonal antibody (as well as sCD4) concentrations greater than 10 µg/ml required to provide 50% virus neutralization is referred to as >10.

### Association of Enhanced Virus Neutralization with Increased Binding of MAbs to Env Trimers and with Low CD4 Dependence

**Figure 6:**
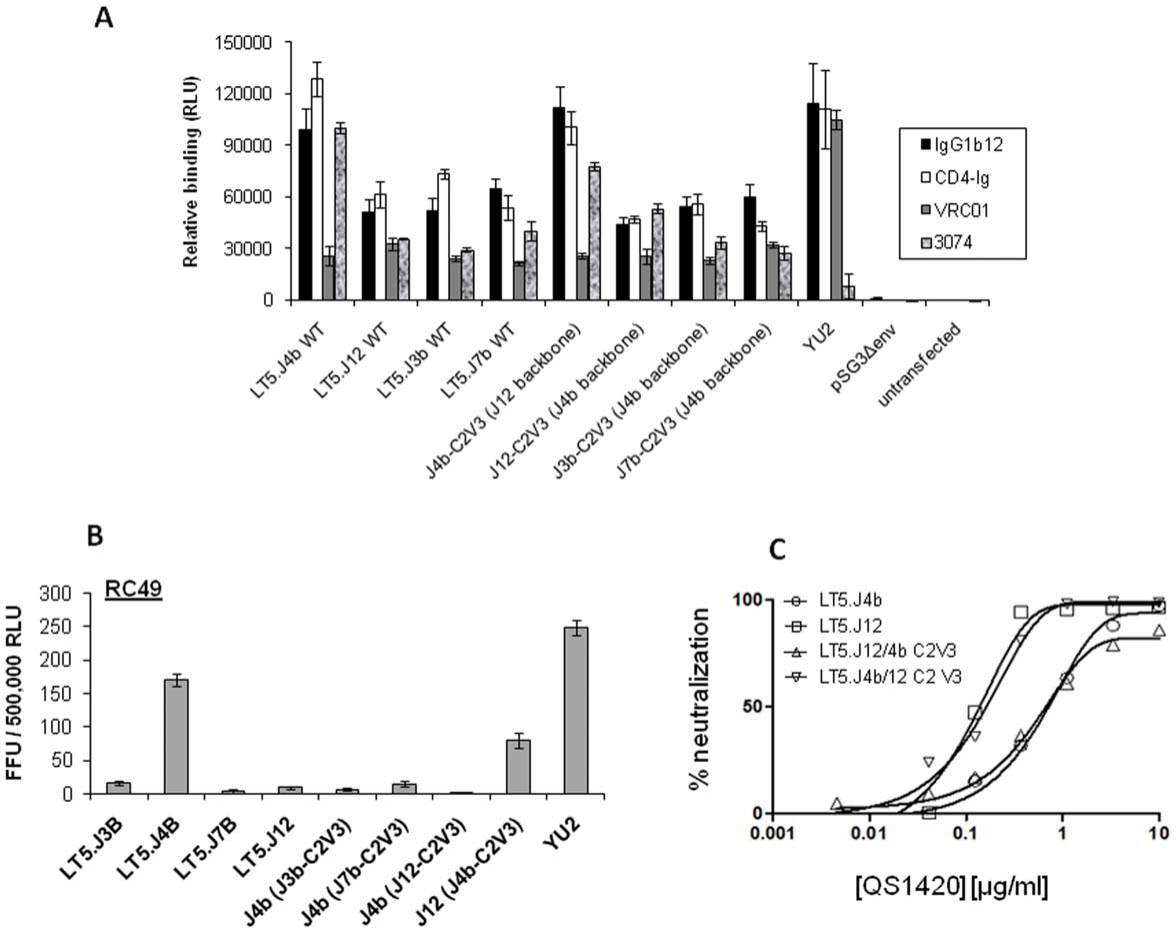
Effect of C2V3 on antibody binding and CD4 dependence (A) Binding of MAbs to envelope trimers expressed on the 293T cells. The binding of ligands was expressed as RLU on the Y-axis for indicated Envs shown on X-axis. The RLU of only pSG3Δenv plasmid was taken as mock and the value was subtracted from the RLU of Env expressing cells. Each ligand was taken as 1 µg/ml for incubation with Env expressing cells for 45 min. The experiments were performed at two different time points (B) Infectivity in HeLa cells (RC49) expressing low CD4 on surface. RC49 cells were infected with equal TZM-bl infectious titer for indicated Envs with YU2 as positive control known to show higher infectivity in RC49. The infection in RC49 cells was measured as Focus Forming Units (FFU) and relative infectivity was in TZM-bl cells were measured and was expressed as FFUs per 5×10^5^ RLU. (C) Inhibition by anti-CD4 (QS1420) MAb. The virus and anti-CD4 was added in the TZM-bl cells to allow them to compete for binding with CD4 on cells. The sensitivity of Envs to QS1420 is shown as percent neutralization of virus on Y-axis in a dose dependent manner.

We next assessed the relationship between presence of unique C2V3 sequence and relative binding of MAbs to Env trimers targeting different regions of Env, which showed enhanced neutralization of LT5.J4b Env. 293T cells were transfected with equal concentrations of Env plasmid DNA and the relative binding of MAbs or CD4-IgG2 to Env trimers expressed on 293T cells was assessed by measuring relative luminescence units (RLU) as described previously [27]. The concentration of each of the MAb that gave 50% neutralization of LT5.J4b was used to examine the Env trimer-MAb interaction. As shown in Figure 6A, the differences in binding were most prominent with IgG1b12 and CD4-IgG2 followed by anti-V3 MAb (3074). However, no difference in binding of Env trimers to VRC01 was observed which correlated with the resistance of the LT5 Envs to VRC01 MAb. Our data indicated that the C2V3 sequence of the LT5.J4b Env altered the overall structure of trimeric Env that favored binding with IgG1b12, CD4-IgG2 and 3074 and was correlated with enhanced neutralization by these reagents.

Since the unique sequence of the C2V3 region was found to modulate both the neutralization sensitivity and binding of Envs to IgG1b12 and CD4-IgG2 which are the prototypic ligands that bind to CD4bs on Env trimer, we subsequently examined the effect of this modulation on the infectivity and CD4 dependence of these Envs. For this, we tested the infectivity of the Envs expressing wild type Envs and C2V3 chimeric Envs in HeLa RC49 cells, expressing high amount of coreceptor CCR5 but limiting amount of CD4 [29]. YU2 was taken as a positive control which is known to be macrophage tropic and has low CD4 dependence for entry. Equal TZM-bl infectious units of Env pseudotyped viruses were taken to infect HeLa RC49 cells and infectivity was measured by immunostaining of p24 positive cells as described before [30]. As shown in Figure 6B, LT4.J4b showed 10.33, 36.83 and 16.63 fold higher infectivity compared to LT5.J3b, J7b and J12 respectively. Interestingly, the infectivity of the LT5.J12 chimeric Env expressing J4b C2V3 showed significant increase in infectivity by 8-folds compared to parental Env; similarly, when C2V3 sequences of LT5.J3b, J7b and J12 were transferred to the LT5.J4b Env, the infectivity of pseudoviruses expressing the LT5.J4b Env was reduced by 26.61, 11 and 79 folds respectively compared to the wild type. The changes in the C2V3 region may impact the crucial binding contacts with the virus receptor and modulate the usage of CD4 or coreceptor for entry. This observation indicating lower CD4 dependence of pseudoviruses expressing the LT5.J4b C2V3 sequences was further substantiated by their increased resistance to anti-CD4 MAb, QS1420 (Figure 6C). Collectively, our data suggested that the C2V3 sequence LT5.J4b Env modulated Env conformation to confer low CD4 dependence for entry and increased binding and neutralization by the MAbs tested here.

### Lack of Association between Neutralization Sensitivity of LT5 Envs with gp120 Shedding

**Figure 7:**
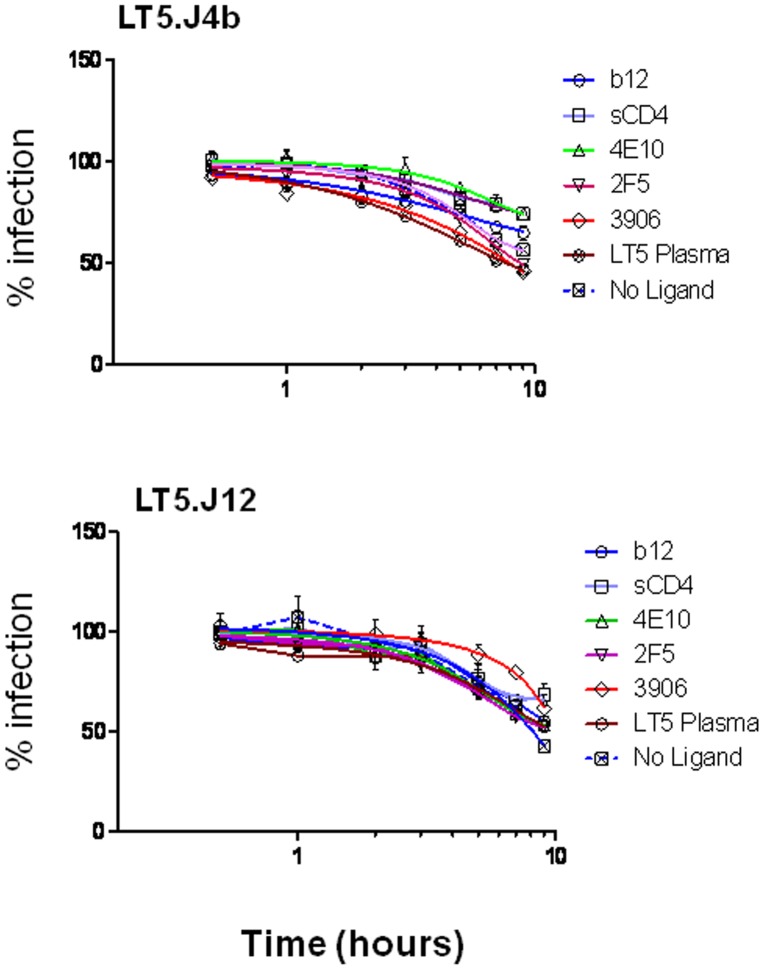
Neutralizing antibody or sCD4 induced shedding of gp120 subunit from Env trimers. Infectivity of the virus treated with a ligand was assessed in TZM-bl cells at different time points to probe the kinetics of MAb or sCD4 neutralization. Ligand concentrations were chosen to yield approximately 50% neutralization after 1 h of pre-incubation of virus with ligand at 37°C to allow for the monitoring of increases in neutralization activity over time. For LT5.J4b which is sensitive 0.02 µg/ml b12, 0.5 µg/ml 4E10, 0.3 µg/ml 2F5, 0.3 µg/ml 3906, 0.5 µg/ml sCD4 and 1∶5000 diluted LT5 plasma whereas for LT5.J12 resistant virus 10 µg/ml b12, 5 µg/ml 4E10, 4 µg/ml 2F5, 10 µg/ml 3906, 10 µg/ml sCD4 and 1∶20 diluted LT5 plasma were taken to treat respective viruses for the indicated time periods at 37°C. Percent neutralization of virus infectivity in TZM-bl cells was calculated with reference to the respective mock-treated virus control of each time point. The experiments were conducted in duplicate at two different time points and one is shown here.

Next we evaluated whether there was any association between neutralization sensitivity of both LT5.J4b (sensitive) and LT5.J12 (resistant) Envs with ligand-induced gp120 shedding. The MAbs and sCD4 (referred to as ligands here) were mixed with pseudotyped viruses expressing LT5.J4b and LT5.J12 Envs with equal infectious TZM-bl titers in a final concentration that showed 50% neutralization of these Envs (see Methods). These virus-ligand mixtures were incubated at 37°C for different time periods as indicated (Figure 7), and the ligand induced gp120 shedding was assessed by measuring the infectivity in TZM-bl cells. As shown in Figure 7, both in case of the LT5.J4b and LT5.J12 Envs, the loss of infectivity at different time points was found to be comparable with the loss observed in absence of any of the ligands. Our data suggested that the enhanced neutralization sensitivity of the LT5.J4b Env due to presence of its unique C2V3 sequence by the MAbs, sCD4 and autologous plasma was primarily due to competitive binding of these ligands to the Env trimers and blocking of its interaction with cellular receptors and was independent of gp120 shedding.

### Antibody Specificity of LT5 Plasma

**Figure 8:**
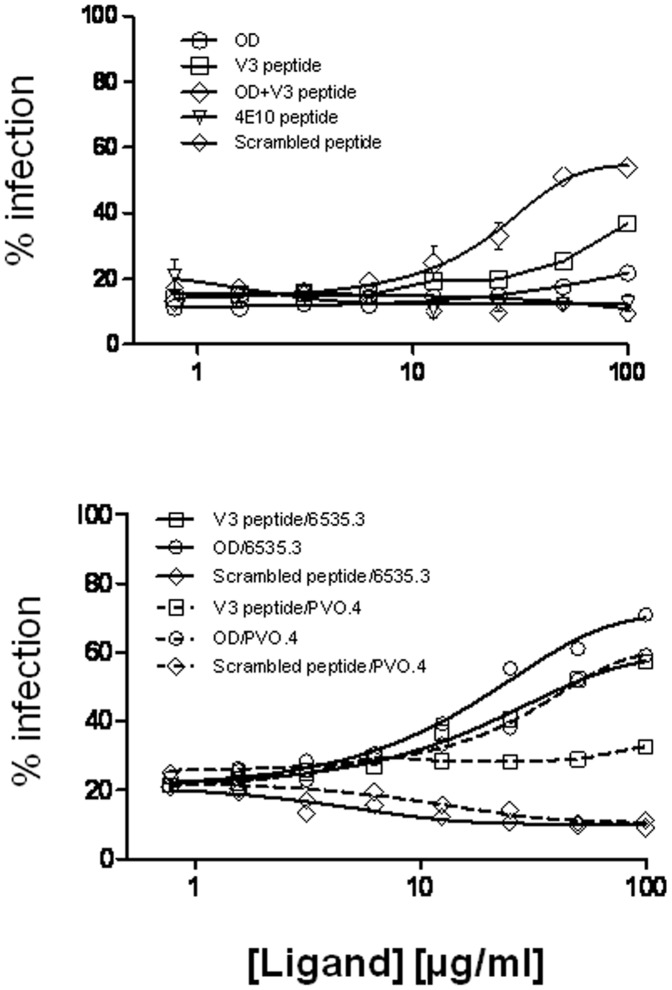
Specificity of the LT5 plasma. LT5 plasma (that showed 80% neutralization of indicated viruses) was incubated with gp120 outer domain (OD) construct, V3 peptide, 4E10 binding peptide and scrambled peptide at various concentrations for 1 hour and subsequently used to assess the neutralizing activity against LT5.J4b and heterologous Env pseudotyped viruses in TZM-bl cells. Assay was done in duplicate and twice. % infection of the viruses on Y-axis was measured as RLU.

Since we found that the LT5.J4b Env that was found to be very sensitive to contemporaneous autologous plasma also showed enhanced sensitivity to IgG1b12 and anti-V3 MAb, the LT5 patient’s plasma showing BCN activity [23] was examined to see if it contained IgG1b12 and/or V3 like antibodies. Thus, to define the antibody specificity, LT5 plasma depleted with the gp120 outer domain protein (devoid of V1V2 and V3 loop sequences) containing 11 mutations at the interface of inner and outer domains to stabilize the CD4 binding site [31] and V3 peptide (based on JRFL sequence containing GPGR motif) and 4E10 binding MPER peptide for 1 hour at 37°C. Prior to adsorption with the probes, the LT5 plasma was diluted in growth medium to yield concentration to provide 80% neutralization of pseudotyped virus expressing LT5.J4b, 6535.3 and PVO.4 Envs. The diluted plasma was subsequently mixed with different concentration of the gp120 outer domain and V3 peptides for 1 hour at 37°C. Along with, LT5.J4b, pseudotyped viruses expressing two clade B Envs (6535.3 and PVO.4) were tested against LT5 plasma pre-adsorbed with the gp120 OD, V3 and MPER peptides. These two clade B Envs were previously found to show neutralization sensitivity to LT5 plasma and were more relevant to test against V3 peptide which is based on clade B Env sequence and contains GPGR motif. [23]. A non-specific peptide having non-natural amino acids was used as negative control. The LT5 plasma pre-adsorbed with the gp120 OD or V3 peptide or 4E10 binding peptides was subsequently incubated with the Env-pseudoviruses (expressing LT5.J4b, 6535.3 and PVO.4 Envs) for 1 hour before adding TZM-bl cells into virus-plasma mixture. As shown in Figure 8, a modest decrease in neutralization of 6535.3 and PVO.4 was noticed with LT5 plasma pre-adsorbed with gp120 OD at a concentration of 50 µg/ml and more. Comparable decrease in neutralization sensitivity of 6535.3 with V3 peptide was noticed but not with PVO.4. Compared to 6535.3 and PVO.4 Envs, the effect of OD and V3 peptide was less on LT5.J4b particularly at the concentrations of peptides that showed greater effects on 6535.3 and PVO.4 Envs. Nonetheless, our data indicated that adsorption of LT5 plasma at higher concentrations of gp120 OD and V3 peptide may provide greater response on LT5.J4b. Interestingly, a greater reduction (by 43%) in the neutralization sensitivity of the LT5.J4b Env was found with the LT5 plasma pre-adsorbed with 1∶1 mixture of gp120 OD and V3 peptides (even though J4b Env contained GPGQ motif in its V3 loop and V3 peptide contained GPGR), further indicating presence of CD4bs and anti-V3 like antibodies in LT5 plasma. Interestingly, the autologous neutralization curve of LT5.J4b found to resemble with the IgG1b12 neutralization curve. Autologous neutralization was found to plateau at 93% while IgG1b12 neutralization at 95% suggests that the LT5 plasma may contain IgG1b12 like antibody and its mechanism of neutralization is similar to IgG1b12. No response was seen with a 4E10 peptide which indicated absence of 4E10 like antibodies in LT5 plasma. The decrease in neutralization susceptibility of the LT5.J4b Env by V3 peptide indicated the presence of clade B and C cross reactive anti-V3 antibodies. Taken together, our data indicated that the BCN activity of LT5 plasma was likely to be due to combined effect of CD4bs and anti-V3 like antibodies. The CD4bs antibody in LT5 plasma however appeared to be different from IgG1b12 as we did not find any significant correlation between the sensitivity of 30 different Envs [23] to IgG1b12 and LT5 plasma (P = 0.36) (Figure S2).

### Structural Variation at IgG1b12 and Anti-V3 (3074) Binding Site between Sensitive (LT5.J4b) and Resistant (LT5.J12) Envs

**Figure 9:**
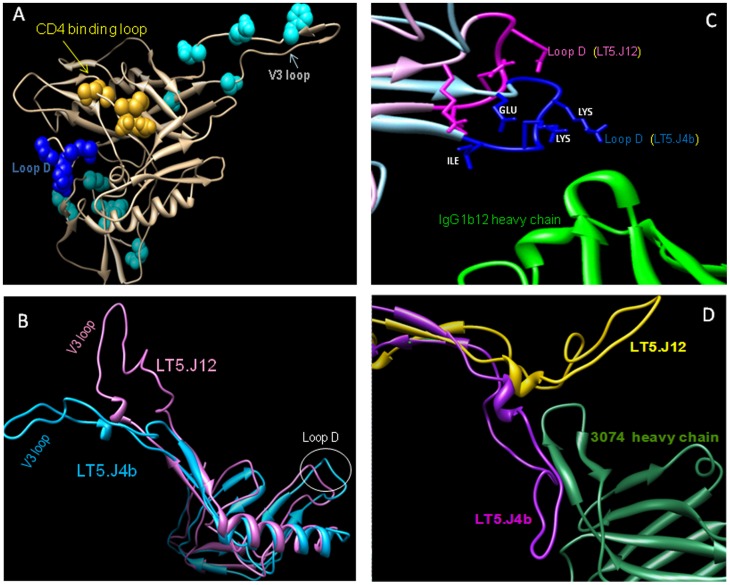
Structure based prediction of the basis of enhanced LT5.J4b Env sensitivity to IgG1b12 (anti-CD4bs) and 3074 (anti-V3) MAbs. A. Amino acid residues in C2V3 region of the LT5.J4b Env that modulated neutralization sensitivity. The model was prepared in UCSF Chimera in the background of JRFL crystal structure (RSCB PDB: 2B4C). The C2V3 amino acid residues that differed between sensitive (LT5.J4b) and other resistant Envs are highlighted. Residues in dark blue are in loop D that contacts CD4 which is in close proximity to major CD4 binding residues (SSGGDPE) (shown in yellow). B Predicated conformations of LT5.J4b and LT5.J12 Env structures in the background of JRFL gp120 crystal structure (2B4C) after energy minimization. C. Spatial orientation of loop D of LT5.J4b and LT5.J12 due to mutations in C2V3 region that showed closer proximity to IgG1b12 heavy chain. D. The interaction of heavy chain of 3074 (green) with V3 loops (LT5.J4b purple, LT5.J12 yellow.

In order to understand if presence of unique mutations in the C2V3 region (Figure 9A) modulated the overall Env conformation, a model of the structure of C2V3 region based on sequences of the sensitive (LT5.J4b) and resistant (LT5.J12) Env was built based on atomic coordinates of HIV-1 gp120 core with variable loops followed by energy minimization in GROMACS. The homology model was built using subtype B gp120 reference structure and so it was possible to have inherent differences in the deduced structure of LT5.J4b and LT5.J12 Envs as significant part (C2V3) of these recombinant Envs is of subtype C. As shown in Figure 9B, significant differences between conformations of LT5.J4b and LT5.J12 Envs were observed. More precisely, IgG1b12 heavy chain formed contacts with loop D residues interact with IgG1b12 and receptor CD4 [32], [33]. When the structure of loop D was compared between the two Envs (Figure 9C), a marked variation in the spatial arrangement of this loop was noticed. LT5.J12 showed a retraction of loop D due to conformational rearrangement exerted by C2 region. The loop D in the LT5.J4b Env relatively was found to be more exposed and protruded from the core to interact with the CDR loops in the IgG1b12 Fab region. The residues in the LT5.J4b Env were within van der wall’s radii of < 7Å of bound antibody heavy chain surface suggesting the improved interaction of IgG1b12 with this LT5.J4b gp120. In addition, presence of unique sequence in the C2V3 region was also found to modulate the V3 loop on gp120 outer domain and the spatial position of V3 loops was markedly varied (Figure 9D). Retraction of C2 region of LT5.J12 resulted in the shift of V3 loop towards gp120 core thus making it less amenable to interact with anti V3 MAb (3074). LT5.J4b showed a deviation of >1.5Å compared to LT5.J12 at the V3 position with minimum change in the CD4 binding site (0.4321Å). Overall, gp120 showed conformational differences with significant root mean square deviation (RMSD) of 0.7432 suggesting that C2V3 region modulated multiple sites in gp120 and that altered the exposure of neutralizing epitopes. Analyses of individual motions in the C2V3 region depicted that the C terminal region of C2 underwent concerted movement (deformation energy of 540) with V3 and in turn, it influenced the movement and the spatial orientation of the V3 loop on the core gp120, thus explaining the effect of mutations in the C2V3 acted in concert and co-modulate the CD4 binding site and the V3 loop.

## Discussion

In the present paper, we examined the vulnerabilities in circulating HIV-1 Envs obtained from plasma of a chronically infected patient that showed cross neutralization of several heterologous HIV-1 Env-pseudotyped viruses. In essence, we examined the determinants in Env that conferred escape from neutralization by autologous antibodies. Although we obtained mixture of clade B and C and B/C recombinants from the plasma of this particular patient, the plasma antibodies exhibited bias towards clade C [23]. Our data indicated neutralization specificity of the LT5 patient’s plasma to CD4 binding sites and V3 loop; in addition a unique sequence pattern of C2V3 region in Env was found to be associated with the increased binding and neutralization of Env-pseudotyped viruses to IgG1b12 (but not VRC01), sCD4 and V3 loop and modestly with 17b targeting CD4i epitopes. The enhanced virus neutralization was further found to be associated with the increased binding of Env trimers with reagents targeting CD4bs and V3 loop and also found to confer low CD4 dependence.

The presence of BCN antibodies in the plasma of the LT5 patient who was chronically infected for several years without any anti-retroviral therapy, possibly accounted for restricting clinical advancement to disease stage. The presence of BCN activity of plasma of the LT5 patient containing mixture of viral subtypes [23] was an observation associated mostly in persistent infections having more viral diversity [4], [7], [34], [35]. One caveat of the study was the unavailability of plasma samples from baseline and earlier time points from this particular patient, which would have helped us to understand the temporal development of neutralizing antibody response, variation in CD4 numbers and more importantly the evolution of circulating Envs. Recently, it was reported that the super infected patients tend to develop broad and potent anti viral immunity than singly infected strain [36], [37]. Similarly, it was possible that the NARI-LT5 patient harboring mixture of subtype B and C variants had driven induction of broadly neutralizing antibodies.

Among the four Envs from LT5 plasma LT5.J4b was found to be most sensitive to autologous plasma and MAbs targeting different regions on Env. Since, we were not able to analyze Env variants from plasma of earlier and later time points; the evolution dynamics of LT5 Envs could not be known. However, the sensitive viral species can emerge *in vivo* even in the pressure of autologous neutralizing activity [38]. Such sensitive Envs might be evolved in the compartment where antibody pressure is lesser and viruses expressing such Env/s may have enhanced replication capacity *in vivo* as suggested by the low CD4 dependence of LT5.J4b Env. Compared to LT5.J4b other Envs exhibited high CD4 dependence for entry and might replicate with slower rate although they are neutralization resistant and possibly might contribute to the survival of sensitive species. The survival of the sensitive viral species could also be the decoy strategy to direct the immune response towards them and to retract the antibody pressure exerted on the resistant species. This phenomenon could possibly be the basis for slow disease progression as far as this particular patient is concerned. Irrespective of the basis of existence, both sensitive and resistant Envs provided an opportunity to dissect the region in the Env that conferred enhanced sensitivity autologous antibodies. The chimeric constructs made by using the LT5.J4b and LT5.J12 Envs revealed that presence of unique C2V3 sequence conferred LT5.J4b Env with enhanced neutralization susceptibility. Though the role of V1V2 in masking the epitopes has been well documented we did not observe any change in neutralization sensitivity to plasma antibodies when this region was transferred from LT5.J4b (sensitive) to LT5.J12 (resistant). The C3 and V4 regions in Env individually or combined was not found to be involved in forming neutralization epitopes. The C3 region contains highly variable α2 helix which was shown by previous studies to modulate the neutralization sensitivity to autologous plasma directly or indirectly by influencing CD4 binding loop in C3 and imparting more flexibility or structural adaptation to the protein in response to neutralizing antibodies [39], [40]. However, in case of LT5 Envs, it appeared that the entire synergistic activity of the mutations in the C2V3 region contributed to modulate the neutralization sensitivity. The coordination of the constant region with other structural elements conserves the core structure and overall function of the Env and this coordination of C2V3 sequence as found in the present study was modulated by the unique mutational pattern imparting specific neutralization phenotype to the LT5 Envs. The C2V3 region contains several β-sheets interspersed with loops: A, B, C and D. While the loop D contains the conserved residues that interact with CD4 and loop B contains residues that contact both CD4 and IgG1b12, any change in this region is expected to directly alter the interaction of gp120 with CD4 or IgG1b12 [32], [33]. Interestingly, replacement of residues in the loop D of LT5.J12 Env with that of the LT5.J4b Env could not restore the neutralization sensitivity of LT5.J12 Env to these ligands. Substitution of the amino acid residues in entire loop D (K275E/E279K/N282K/T283I) together or individually did not alter the sensitivity of LT5.J12 Env (Table 1). Loop B was conserved in all four functional LT5 Envs but two changes one downstream within the bridge between β6 and β7 (K240) and another in β8 (Q246) were present in the LT5.J4b Env. Substituting these residues (Q240K and K246Q) in the resistant LT5.J12 Env marginally modulated its neutralization sensitivity to sCD4, IgG1b12 and autologous plasma antibodies (Table 1). Other substitutions either in loop A or C-terminal region of C2 forming β12 which also contains an additional N-linked glycan site did not modulate the neutralization sensitivity (Table 1). Recently, Utachee *et al* [41] demonstrated that the removal of glycans in the V2 and C2 regions increases the IgG1b12 sensitivity of HIV-1 CRF01_AE glycoproteins. In our study, removal of extra glycans from V2 [27] and C2 regions (such as N289D) had no effect on overall sensitivity of Envs to IgG1b12. Nonetheless, it highlights the importance of this region (C2V3) in modulating the sensitivity to CD4bs directed antibodies and provide additional site of vulnerability on Env.

The sequence conservation of the C2V3 region and that it contains the CD4 contact residues signifies the functional role of this region [32], [33]. CD4 binds to the major groove in the gp120 made between outer and inner domain and another major binding surface is provided by the Phe43 cavity that is itself present at the interface between the outer and inner domain. Within C2 region, residues such as V255 and T257 are important that line the Phe43 cavity among others [32]. Although these residues were conserved in all the NARI-LT5 Env clones [23], it is possible that the spatial arrangement of these residues was influenced by the proximal residues. The residues proximal to CD4 binding loop take part in receptor binding and impact infectivity in low CD4 expressing cells due to their role in conformational changes at CD4 binding site thereby allowing better access and higher affinity [42], [43] The variation proximal to the conserved CD4 contacting residues vindicates the role of these residues in preserving the CD4 binding loop from antibodies by providing interactive surface mismatch while maintaining its primary function. The V3 loop and CD4bs elements are thought to be in proximity and changes within these regions can mutually alter the sensitivity to the ligands directed to those sites [42]. The transfer of the V3 loop from the LT5.J4b Env to LT5.J12 Env did not change the sensitivity of LT5.J12 Env to IgG1b12 or anti-V3 MAbs but the entire C2V3 region substantially enhanced its sensitivity to IgG1b12, sCD4, anti-V3 MAb (3074) as well as 17b MAb, suggesting that the conformation of the neutralization competent structures (or their exposure) made by V3, CD4 binding loop D and coreceptor binding residues on unliganded gp120 was likely co-modulated by elements in the C2 and V3 regions. However, unlike CD4 usage, no alteration in CCR5 usage of LT5 Envs was found suggesting that coreceptor binding sites exposure on gp120 post CD4 binding was similar but it substantially varied when gp120 was not engaged by CD4, evident from 17b MAb binding to Env trimers expressed on 293T cells. Our study also highlighted presence of a rare substitution in the V3 loop (I315F) in the LT5.J4b Env (but absent in other LT5 Envs) which was previously shown to modulate susceptibility of HIV-1 Envs to anti-V3 MAbs [42], [44]. Interestingly, the determinants in Env found to modulate the sensitivity of HIV-1 to IgG1b12 or sCD4 did not show any impact on susceptibility to the VRC01 MAb; a broad and a very potent CD4bs neutralizing MAb [16]. In fact, all the four NARI-LT5 Envs showed resistance to VRC01 MAb, suggesting that the different set of determinants was involved with Env interaction that differs from recognition of IgG1b12 [45].

The glycosylation on the Env trimer is another important factor that influences the neutralization sensitivity. Presence of a PNG at the 386 position shields the neutralizing epitopes in the CD4bs and its removal was previously shown to increase the sensitivity of HIV-1 Envs to IgG1b12 apparently by filling a cavity penetrated by W100 of IgG1b12 [46], [47], [48]. In this study, although LT5.J4b Env lacking PNG N386 was found to be sensitive to b12 MAb, however, when we transferred the C3V4 segment of the LT5.J4b Env to the resistant LT5.J12 Env, we did not find any modulation in its sensitivity to IgG1b12 MAb, suggesting that N386 did not play any significant role in LT5 Envs, possibly due to the presence of other compensatory determinants. In addition, N283 glycan in the C2 region, which was found to be absent in all the LT5 Envs, was previously shown to enhance the macrophage infectivity as well as enhanced sensitivity to IgG1b12 [46], [49]. Nonetheless, despite lack of N283, we found low CD4 dependence of the LT5.J4b Env, thus highlighting role of other compensatory determinants conferring efficient infection of low CD4 expressing cells.

Our data provided important information on atypical determinants in the C2V3 region of Env that gave rise to greater exposure of neutralizing epitopes in CD4bs, V3 loop and coreceptor binding sites. Both CD4bs and V3 loop are important regions on gp120 which are highly antigenic and immunogenic in nature [50], [51]. In addition, the CD4bs and V3 specific neutralizing antibodies have been shown to display potent and cross-clade neutralizing activity [6], [21], [50], [52], [53], [54], [55], [56]. The determinants are located in C2 region away from the main CD4 binding loop but also involve residues in the V3 loop. An Env form such as the one we found here (LT5.J4b) might be a preferred version to be recognized by immune cells and elicit the specific antibodies against functionally conserved CD4bs and proximal epitopes in V3 and its base. Recently, it has been shown that the stabilization of CD4bs by cavity filling mutations and deletions of variable loops elicited neutralizing antibodies targeting CD4bs and similarly to coreceptor binding sites [50], [57]. Although it remains to be tested whether breadth and potency developed in LT5 plasma was due to LT5.J4b Env like immunogens; the determinants in C2V3 region indeed provided an important insight towards the exposure of functionally conserved epitopes. Another limitation of the study is that the assays were performed with env pseudotyped viruses prepared in 293T cells. It may be possible that the full length versions from PBMCs display different gp160 glycoform which may impact the results found here. However, others have found that PBMC-derived viruses and pseudoviruses with the same Env protein give qualitatively similar results [58].

Although the specificity of LT5 plasma was partially mapped to CD4bs and V3 loop it is possible that other specificities were also present as the gp120 OD construct used to adsorb IgG1b12 like antibodies may also adsorb antibodies which bind further apart from CD4bs on gp120 outer domain. Multiple antibody specificities were reported in chronic infections [1], [2], [3], [4], [5], [6] and our observation of association between specificities of the LT5 plasma to IgG1b12 and V3 like antibodies and exposure of neutralizing epitopes in the CD4bs and V3 loop of LT5.J4b Env provided important insights in HIV-1 Env that may inform rational Env-based vaccine design.

## Methods

### Construction of Chimeric and Mutant Clones

Chimeric Env cloning and site-directed mutagenesis was carried out by using tailor-made primers as shown in Figure S3. To transfer a region from donor plasmid to a recipient plasmid the overlapping primer pairs having at least 15 base pair homology were developed that anneal to the conserved region flanking the env domain to be transferred. One primer pair amplified the insert from a donor plasmid and another pairs which amplified backbone of recipient plasmid away from the corresponding region. These PCR products were first digested with DpnI enzyme for 1 hour to remove the methylated template plasmid and then purified by PCR purification kit (Qiagen Inc.). Purified PCR products having at least 15 base pair homology at both the ends were treated then annealed after the treatment with pox DNA polymerase provided in Dry Down PCR Cloning kit (Clontech Inc.) following manufacturer’s protocol and as described previously [59]. The cocktail of annealed PCR fragments was directly transformed in *E coli* competent cells. Similar procedure was followed for site directed mutagenesis except the forward primer of insert and corresponding backbone primer which amplifies in opposite direction introduced single or multiple mutation.

### Neutralization Assay

The neutralization assays were carried out as described earlier [59], [60]. Briefly, Env-pseudotyped viruses were incubated with different concentrations of MAbs or sCD4 for 1 hour at 37°C, following which 1×10^4^ TZM-bl cells were added into the mixture in presence of DEAE-Dextran (Sigma, Inc.) making final concentration 25ug/ml (Sigma Inc.). The plates were incubated in a CO_2_ incubator for 48 hours and the degree of virus neutralization was assessed by measuring relative luminescence units (RLU) by using Britelite^TM^ luciferase substrate (Perkin Elmer Inc.) in a luminometer (Victor 4, Perkin Elmer Inc.).

### Cell-based Binding Assay

The relative interaction of Env with MAbs or CD4-IgG2 was assessed by cell-based binding assay as described before [27], [61]. Briefly, 293T cells were transfected with equal amounts of Env plasmids and at 48 hours post-transfection, cells were washed to remove growth media following which MAb or CD4-IgG2 was added at 5ug/ml concentrations to allow binding with env^+^ 293T cells for 1 hour at 37°C and subsequently with goat-anti-human IgG conjugated with horse radish peroxidase (HRP; Thermo Scientific, Inc.). The cells were washed for four times with first blocking buffer (35 mg/ml BSA, 10 mg/ml non-fat dry milk, 1.8 mM CaCl_2_, 1 mM MgCl_2_, 25 mM Tris, pH 7.5 and 140 mM NaCl) [62] and subsequently with washing buffer (140 mM NaCl, 1.8 mM CaCl_2_, 1 mM MgCl_2_, 20 mM Tris, pH 7.5) [62] after each incubation with primary and HRP-conjugated secondary antibodies. The relative binding of Env with ligand was assessed by addition of luminol substrate (Thermo Scientific, Inc) and measuring RLU in luminometer.

### gp120 Shedding Assay

The gp120 shedding was assessed by measuring infectivity of virus incubated at 37°C as described by Ruprecht *et al.* [63]. Briefly, a fixed virus input (2×10^5^ RLU/well); no MAbs) was incubated for the indicated time periods at 37°C in culture medium supplemented with a ligand in the concentration that gives approximately 50% neutralization at 1 hour of incubation. The virus incubated at different time points was then used to infect TZM-bl target cells, and virus infectivity was monitored by luciferase activity at 72 h after infection. Time of addition experiments were conducted to probe the kinetics of virus neutralization by different ligands and to assess the induction of gp120 shedding from the virions. Antibody concentrations were chosen to yield neutralization of approximately 50% after 1 h of pre-incubation to allow for the monitoring of increases in neutralization activity over time. The selection of precise ligand concentration was possible for LT5.J4b as it is sensitive to most ligands tested here but LT5.J12 was resistant at 10 ug/ml, highest concentration tested. For LT5.J12 therefore, the highest concentration was used to assess the gp120 shedding over the period of time.

### Adsorption of LT5 Serum

Adsorption of LT5 plasma was performed by using an outer domain (OD) protein of gp120 [31], JRFL based V3 peptide (V3B-3: KSIHIGPGRAFYTTG; kindly provided by Prof Raghavan Varadarajan, Indian Institute of Sciences, Bangalore, India), 4E10 peptide (NH2-CWNWFDITKWLWYIKKKK-CONH2) and a scrambled peptide (P6: NH2-C-W-delF-W-F-D-delF-T-K-W-L-W-Y-I-K-K–CONH2) which is a 4E10 nonbinding peptide wherein N671 and I675 has been replaced with non-natural amino acid dehydrophenylalanine. The gp120 OD is *Escherichia coli*-expressed protein based on the sequence of the HxBc2 strain which binds CD4 and the broadly neutralizing antibody IgG1b12 but not the non-neutralizing antibodies b6 and F105 [31]. LT5 plasma was diluted in growth medium to yield 80% neutralization (20% infection compared to virus control) of the indicated virus after 1 hour of incubation and dispensed in 96 well plate. The OD and V3 constructs were serially diluted in LT5 plasma suspension starting from 100 µg/ml in two fold dilutions and plate was incubated for 1 hour at 37°C in order to adsorb the IgGs in LT5 plasma onto the probes. 1.5×10^5^ RLU virus was then added in each well and incubated the plate for another 1 hour. After incubation 1×10^4^ cells were added with 25 ug/ml dextran and incubated the plate for 48 hours. Infection of TZM-bl cells was measured by luciferase activity and the increase in infection in presence of probe was compared with the wells where probe was not added.

### Statistical Analysis

The correlations between IgG1b12 and LT5 plasma sensitivities of a panel of 30 Envs [23] were evaluated for statistical significance by Spearman test. Correlation co-efficient expressed as Spearman’s rho (r) and P values shown at the top of each box were obtained by using Graph Pad prism software.

### Molecular Modeling

Structural models of HIV-1 C2V3 regions were produced with SWISS MODEL homology modeling server in project mode [64], using HIV-1 JRFL gp120 (PDB: 2B4C) [65], b12 (PDB: 2NY7) [33] and anti-V3 MAb 3074 (PDB: 3MLZ) (Jiang et al., 2010 [66] crystal structures. Additional energy minimization was carried out using GROMACS with L-BFGS (Low-memory Broyden-Fletcher-Goldfarb-Shanno quasi-Newtonian minimizer) method that approximates an inverse Hessian matrix from previous configurations [67] and was more suitable for analyses of concerted C2V3 movements. The final minimized structures were superposed and visualized in UCSF Chimera [68].

### Competing Interests

The authors declare that they have no competing interests.

## Supporting Information

Figure S1 **Neutralization sensitivity of LT5 Envs to VRC01 and 2F5 monoclonal antibodies.** Note that all the LT5 Envs showed resistance to VRC01 MAb including the sensitive LT5.J4b Env.
(TIF)

Figure S2 **Correlation between IgG1b12 and LT5 plasma sensitivities of a panel of 30 Env pseudotyped viruses** [**23**] **.**
(TIF)

Figure S3 **Construction of chimeric and mutant Envs.** (A) Chimeric Envs were constructed using a region exchange strategy and mutant Envs were made by introducing specific nucleotide change in primer. (a) The domain to be transferred for example C2V3 (light blue box) was PCR amplified from the env gene using primers (arrows) that annealed to conserved sequences flanking C2V3 and amplified inward. (b) The recipient Env (open box) plus plasmid vector was PCR amplified using primers (arrows) that annealed to sites adjacent but overlapping (at least 15 base pairs) to those of the C2V3 primers and amplified outward. (c) The two fragments were then mixed with PCR cloning cocktail containing pox virus DNA polymerase which forms cohesive ends in PCR fragments by its 3′–5′ exonuclease activity. The cohesive ends anneal and forms circular plasmids with a nick at each strand. The annealed product is directly transformed in competent cells where the nicks are sealed. (B) The PCR fragments after treatment with dry down cloning cocktail. The homologous sequence is annealed to yield circular plasmid. (a) chimeric and (b) mutant Env preparation is shown.
(TIF)
