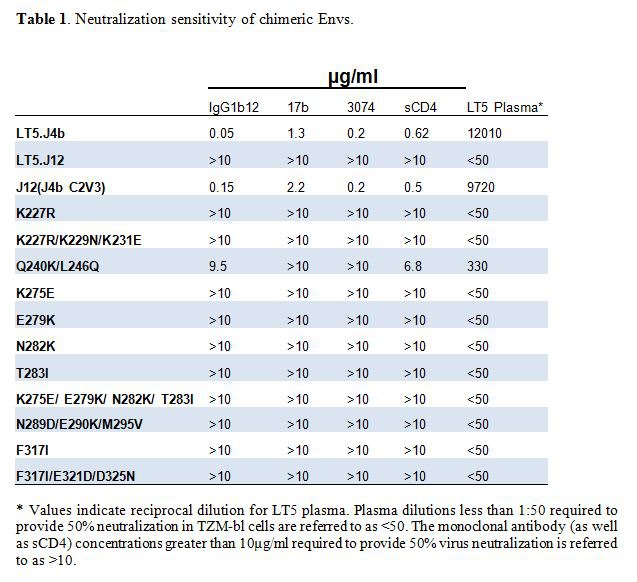# Correction: Unique C2V3 Sequence in HIV-1 Envelope Obtained from Broadly Neutralizing Plasma of a Slow Progressing Patient Conferred Enhanced Virus Neutralization

**DOI:** 10.1371/annotation/6168e62e-6d5b-47a4-bf92-bae95d09d92b

**Published:** 2013-10-10

**Authors:** Rajesh Ringe, Lipsa Das, Ipsita Choudhary, Deepak Sharma, Ramesh Paranjape, Virander Singh Chauhan, Jayanta Bhattacharya

There was an error in the headings for Table 1. The correct Table 1 can be viewed here: 

**Figure:**