# Supplementary figures and images for: Unique C2V3 Sequence in HIV-1 Envelope Obtained from Broadly Neutralizing Plasma of a Slow Progressing Patient Conferred Enhanced Virus Neutralization
Source: PLoS One. 2012 Oct 3;7(10):e46713. doi: 10.1371/journal.pone.0046713 (PMC3463516; doi:10.1371/journal.pone.0046713)

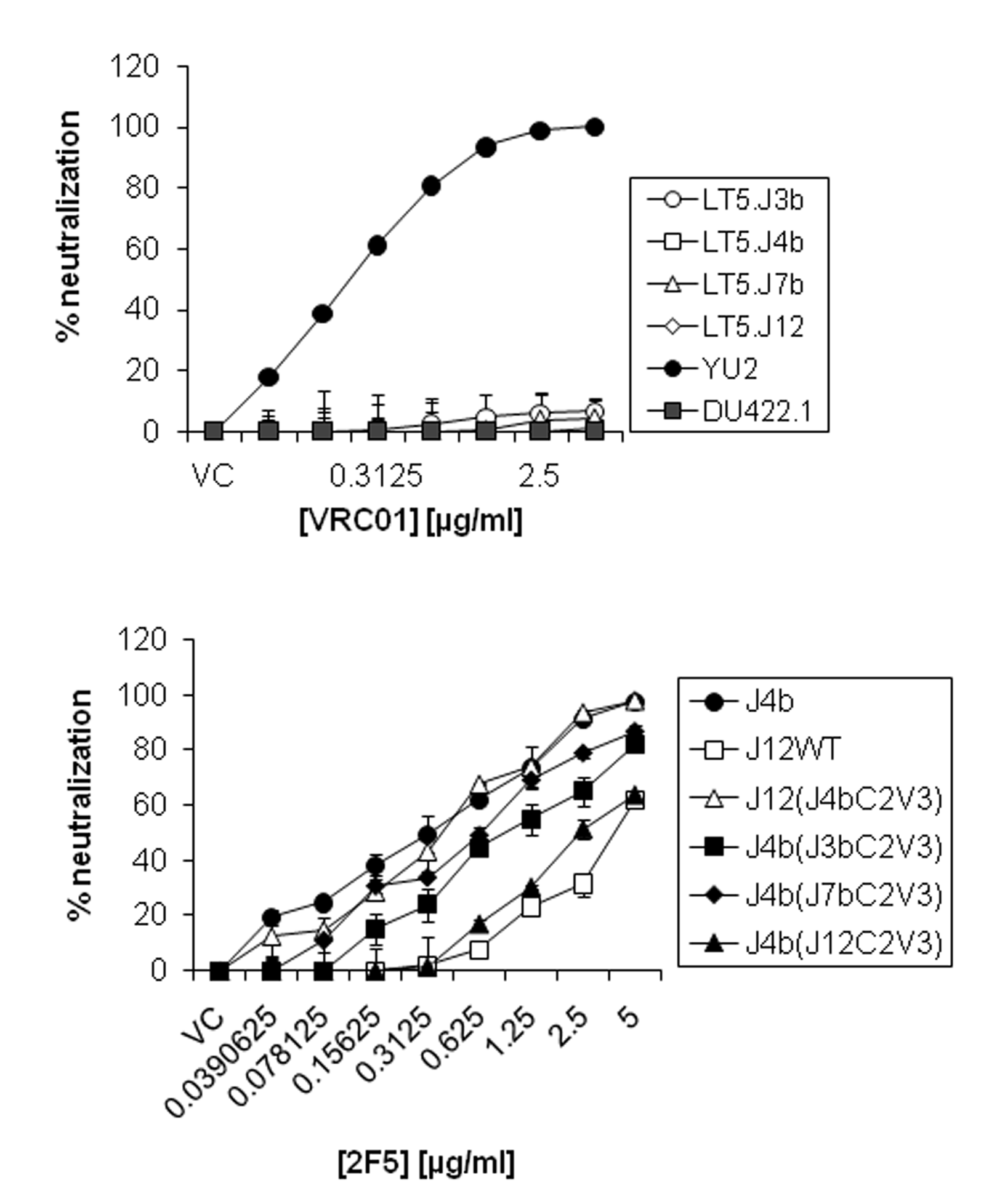

Supplement: Figure S1 — Neutralization sensitivity of LT5 Envs to VRC01 and 2F5 monoclonal antibodies. Note that all the LT5 Envs showed resistance to VRC01 MAb including the sensitive LT5.J4b Env. (TIF) [file pone.0046713.s001.tif]

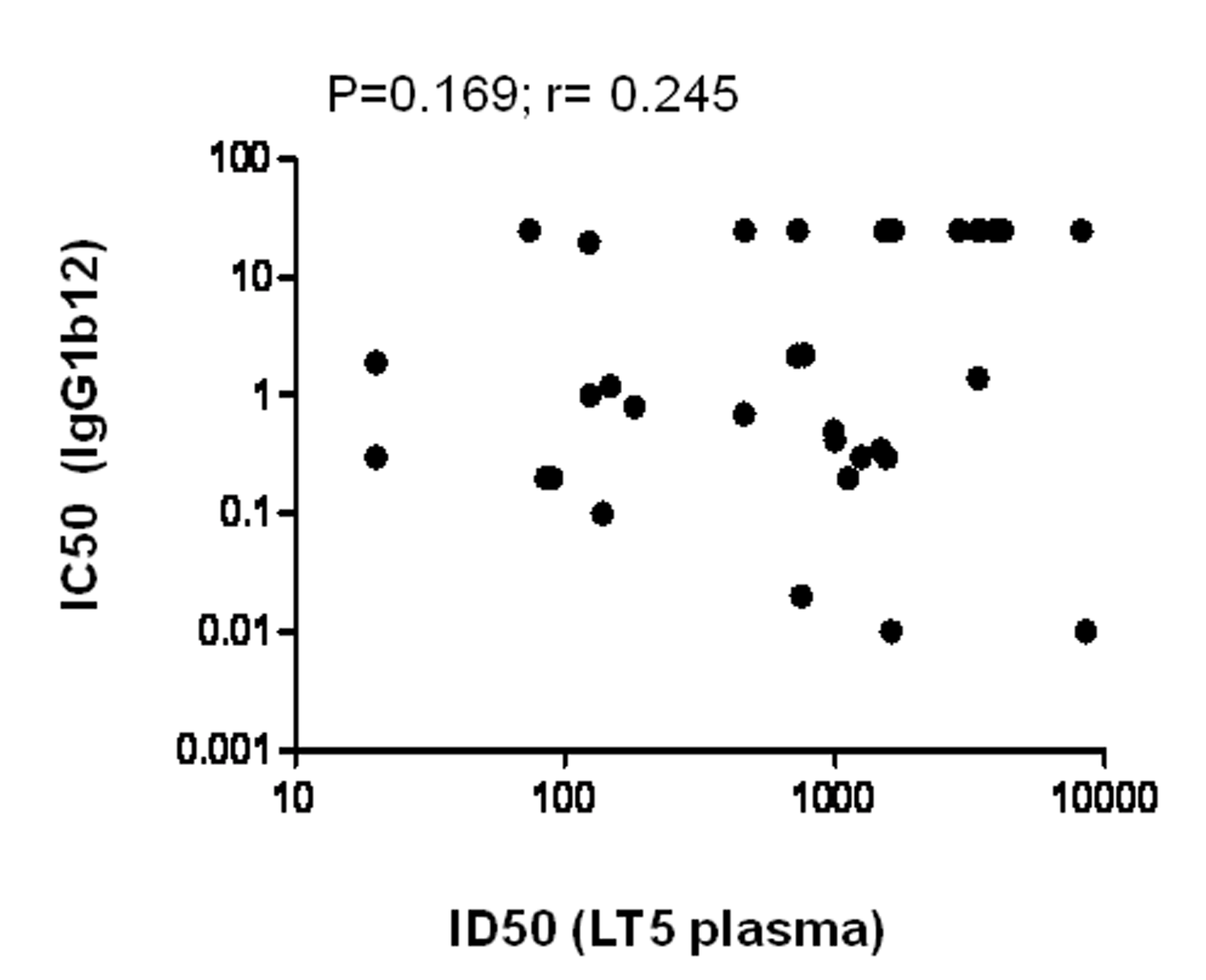

Supplement: Figure S2 — Correlation between IgG1b12 and LT5 plasma sensitivities of a panel of 30 Env pseudotyped viruses [23] . (TIF) [file pone.0046713.s002.tif]

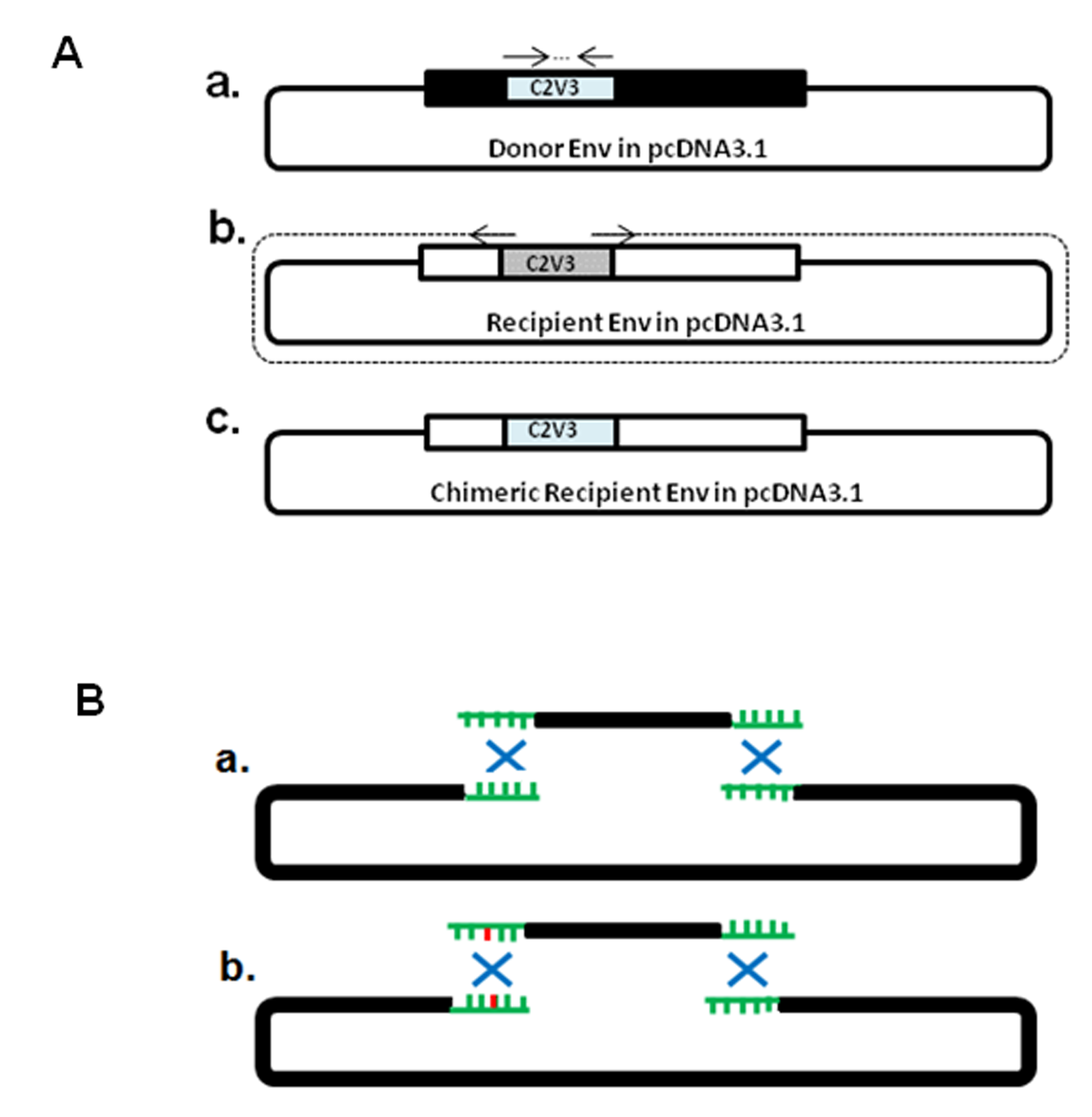

Supplement: Figure S3 — Construction of chimeric and mutant Envs. (A) Chimeric Envs were constructed using a region exchange strategy and mutant Envs were made by introducing specific nucleotide change in primer. (a) The domain to be transferred for example C2V3 (light blue box) was PCR amplified from the env gene using primers (arrows) that annealed to conserved sequences flanking C2V3 and amplified inward. (b) The recipient Env (open box) plus plasmid vector was PCR amplified using primers (arrows) that annealed to sites adjacent but overlapping (at least 15 base pairs) to those of the C2V3 primers and amplified outward. (c) The two fragments were then mixed with PCR cloning cocktail containing pox virus DNA polymerase which forms cohesive ends in PCR fragments by its 3′–5′ exonuclease activity. The cohesive ends anneal and forms circular plasmids with a nick at each strand. The annealed product is directly transformed in competent cells where the nicks are sealed. (B) The PCR fragments after treatment with dry down cloning cocktail. The homologous sequence is annealed to yield circular plasmid. (a) chimeric and (b) mutant Env preparation is shown. (TIF) [file pone.0046713.s003.tif]
